# Midlife Plasma Aβ and Late-Life Risk of Cognitive Impairment: The Atherosclerosis Risk in Communities Study

**DOI:** 10.1093/geroni/igaa057.810

**Published:** 2020-12-16

**Authors:** Kevin Sullivan, Chad Blackshear, A Richey Sharrett, Rebecca Gottesman, David Knopman, B Gwen Windham, Michael Griswold, Thomas Mosley

**Affiliations:** 1 University of Mississippi Medical Center, Jackson, Mississippi, United States; 2 Johns Hopkins University, Baltimore, Maryland, United States; 3 Johns Hopkins School of Medicine, Baltimore, Maryland, United States; 4 Mayo Clinic, Rochester, Minnesota, United States; 5 The University of Mississippi Medical Center, Jackson, Mississippi, United States

## Abstract

Plasma-based biomarkers of amyloid beta (Aβ), a neuropathological hallmark of Alzheimer’s disease, show promise in predicting cognitive impairment and mapping onto cerebral amyloidosis, but little is known about how midlife plasma Aβ associates with late-life cognitive outcomes. Midlife plasma variants Aβ42 and Aβ40 were measured using a fluorimetric bead-based immunoassay in a subsample of visit 3 ARIC participants (1993-95; n=2585, mean age=59.4±5.2, 57% female, 23% African American). We investigated the relationship between midlife plasma Aβ and late-life mild cognitive impairment (MCI; n=923) and dementia (n=628) diagnosed from 2011-19. Multinomial logistic regressions estimated relative risk ratios (RRR) of MCI, dementia, and death vs normal cognitive status as a function of:(1) Aβ42:Aβ40 ratio, (2) Aβ42 and Aβ40 included as separate terms, and (3) Protected Aβ group (participants with Aβ42≥46 pg/ml and Aβ40 <233 pg/ml). Adjusters included age, sex, education, site-race, and APOE4. Every doubling of midlife plasma Aβ42:Aβ40 up to a threshold of 0.20 was associated with 41% lower risk of developing MCI/dementia in comparison to cognitively normal (RRR=0.59 [95% CI:0.42, 0.82]), with no association for ratio values ≥0.20. Every standard deviation increase in plasma Aβ42 was associated with 17% lower risk of dementia (RRR=0.83 [0.70, 0.99]), whereas every standard deviation increase in plasma Aβ40 was associated with 16% higher risk of MCI (RRR=1.16 [1.02, 1.31]). The protected midlife plasma Aβ group had 86% lower risk of late-life dementia vs all others (RRR=0.14 [0.04, 0.47]). Early measurement of plasma Aβ may prove an accessible and effective population screener for future cognitive impairment.

